# Genome-Wide Identification and Expansion Patterns of SULTR Gene Family in Gramineae Crops and Their Expression Profiles under Abiotic Stress in *Oryza sativa*

**DOI:** 10.3390/genes12050634

**Published:** 2021-04-23

**Authors:** Zhengqing Yuan, Weixiong Long, Haifei Hu, Ting Liang, Xiaoyun Luo, Zhongli Hu, Renshan Zhu, Xianting Wu

**Affiliations:** 1 State Key Laboratory of Hybrid Rice, Key Laboratory for Research and Utilization of Heterosis in Indica Rice of Ministry of Agriculture, Engineering Research Center for Plant Biotechnology and Germplasm Utilization of Ministry of Education, College of Life Science, Wuhan University, Wuhan 430072, China; 13971514338@163.com (Z.Y.); weixionglong@whu.edu.cn (W.L.); TingL19930920@126.com (T.L.); luoxy0916@whu.edu.cn (X.L.); huzhongli@whu.edu.cn (Z.H.); renshan8@whu.edu.cn (R.Z.); 2 School of Biological Sciences, University of Western Australia, Perth 6009, Australia; haifei.hu@research.uwa.edu.au

**Keywords:** *Oryza sativa*, sulfate transporter, comparative analysis, gene duplication, Gramineae crops

## Abstract

Sulfate transporters (SULTRs), also known as H^+^/SO_4_^2−^ symporters, play a key role in sulfate transport, plant growth and stress responses. However, the evolutionary relationships and functional differentiation of SULTRs in Gramineae crops are rarely reported. Here, 111 SULTRs were retrieved from the genomes of 10 Gramineae species, including *Brachypodium disachyon*, *Hordeum vulgare*, *Setaria italica*, *Sorghum bicolor*, *Zea mays*, *Oryza barthii*, *Oryza rufipogon*, *Oryza glabbermia* and *Oryza sativa* (*Oryza sativa* ssp. indica and *Oryza sativa* ssp. japonica). The SULTRs were clustered into five clades based on a phylogenetic analysis. Syntheny analysis indicates that whole-genome duplication/segmental duplication and tandem duplication events were essential in the SULTRs family expansion. We further found that different clades and orthologous groups of SULTRs were under a strong purifying selective force. Expression analysis showed that rice SULTRs with high-affinity transporters are associated with the functions of sulfate uptake and transport during rice seedling development. Furthermore, using *Oryza sativa* ssp. indica as a model species, we found that *OsiSULTR10* was significantly upregulated under salt stress, while *OsiSULTR3* and *OsiSULTR12* showed remarkable upregulation under high temperature, low-selenium and drought stresses. *OsiSULTR3* and *OsiSULTR9* were upregulated under both low-selenium and high-selenium stresses. This study illustrates the expression and evolutionary patterns of the SULTRs family in Gramineae species, which will facilitate further studies of SULTR in other Gramineae species.

## 1. Introduction

Sulfur is the least abundant macronutrient and plays an essential role in plant growth and response to various stresses [1,2]. In higher plants, sulfur is predominantly acquired from the soil in the form of anionic sulfate (SO_4_^2−^) mediated by plasma membrane-localized H^+^/SO_4_^2−^ co-transport process [3,4,5]. Various transporters, including the plasma membrane sulfate transporter, ATP-dependent sulfate and thiosulfate transporter, have evolved in plants for transportation of the sulfate [6]. These transporters are responsible for the absorption and transport of the anionic sulfate, which is associated with plant yield, stress responses and environmental adaption [7,8].

Sulfate transporters (SULTRs), which contain 12 *trans*-membrane domains and one sulfate transport anti-sigma domain (STAS) at the *C*-terminal region, are known as H^+^/SO_4_^2−^ symporters and are located on the plasma membrane [9]. SULTRs have been reported to be involved in sulfate transport and are well studied in *Arabidopsis thaliana* [10]. The reported SULTRs are classified into four groups according to their affinity [11]. The high-affinity transporters *AtSULTR1*;*1* and *AtSULTR1*;*2* co-localized in roots, function in soil sulfate uptake [12]. Another high-affinity *AtSULTR1*;*3* is localized in the phloem and participates in transport of sulfate. Low-affinity transporters (*AtSULTR2*;*1* and *AtSULTR2*;*1*) mediate long-distance transport of sulfate in the vascular tissue. To date, the roles of the third group of SULTRs in *Arabidopsis* remain unclear. The last group of transporters (*AtSULTR4*;*1* and *AtSULTR4*;*2*), localized in the tonoplasts, function in the efflux of sulfate from the vacuolar space into the cytoplasm [13]. In addition to the functional analysis of the SULTRs in *Arabidopsis*, several studies of SULTRs have been conducted in major crops, such as maize, rice, wheat, *Brassica oleracea* and soybean [1,11,13,14]. A total of 14 SULTRs have been identified in the *Oryza sativa* ssp. indica genome with varying expression patterns [15]. According to the microarray analysis, *OsSULTR1*;*1*, *OsSULTR1*;*2*, *OsSULTR2*;*1*, and *OsSULTR5*;*2* showed high expression in the roots of seedlings [16]. Further, the sulfur status of the plant has a significant impact on the expression of sulfate transporters under biotic and abiotic stress [11,17]. The mutation of *OsSULTR3*;*3* contributes to altering the accumulation of phosphate and sulfate in rice seedlings [18]. Additionally, a recent study reported that SULTRs in maize function in adaptation to sulfur deficiency conditions [11].

Although SULTRs have been characterized in maize, rice and *Arabidopsis*, in-depth studies are lacking in other Gramineae plants, which limits further understanding of the expansion pattern of the SULTRs in Gramineae. Gramineae species represent major food crops with high economic and industrial value. High-quality genome sequences are available for these species, and their phylogeny has been well-studied [19,20]. This makes the Gramineae an ideal model system for studying the evolutionary dynamics of the SULTR family.

In this study, the SULTRs families from 10 Gramineae species, including *Brachypodium distachyon*, *Hordeum vulgare*, *Oryza barthii*, *Oryza glabbermia*, *Oryza rufipogon*, *Oryza sativa* ssp. japonica, *Oryza sativa* ssp. indica, *Sorghum bicolor*, *Setaria italica* and *Zea mays* were identified. To investigate the gene expansion and evolution patterns of SULTRs, we conducted several analyses related to phylogeny, chromosomal distribution, gene structure, protein motif, orthogroups, duplication events, selective forces, expression profiles and comparative genetics. Additionally, we analyzed the expression patterns of SULTRs under various stress conditions, including salt, heat, drought, low-selenium and high-selenium stress. Our results will facilitate further investigations of SULTRs in Gramineae.

## 2. Materials and Methods

### 2.1. Identification and Phylogenetic Analysis of SULTRs

The complete genome sequences and protein sequences of 9 Gramineae species, comprising *Brachypodium distachyon* (v3.0), *Hordeum vulgare* (IBSC_v2), *Oryza barthii* (v1), *Oryza glabbermia* (v1), *Oryza rufipogon* (OR_W1943), *Oryza sativa* (IRGSP-1.0), *Setaria italica* (v2.0), *Sorghum bicolor* (NCBIv3) and *Zea mays* (B73_RefGen_v4), were downloaded from Ensembl Plant release 48 (https://plants.ensembl.org/index.html, accessed on 20 March 2021). The cDNA and protein sequences of another subspecies of Asian cultivated rice, *Oryza sativa* ssp. indica (Osi, R498), were downloaded from http://mbkbase.org/R498/, accessed on 20 March 2021). The Sulfate_transp domain (PF00916) was obtained from the Pfam database (https://pfam.xfam.org/, accessed on 20 March 2021). The SULTRs were identified in the 10 genomes using HMMER v3.3.1 (http://hmmer.org, accessed on 20 March 2021) with a cut-off e-value threshold of 1 × 10^−^^10^ and BLASTP v2.9.0 with an e-value threshold of 1 × 10^−^^20^ [20,21]. The protein sequences of primarily identified SULTRs with a length of >330 aa were selected for further analysis. The longest transcript of each SULTR was retained. To further validate the SULTRs, the identified SULTR protein sequences of each species were searched against known conserved domain models using Batch CD-Search (https://www.ncbi.nlm.nih.gov/Structure/bwrpsb/bwrpsb.cgi, accessed on 20 March 2021) [22]. All protein sequences of the identified SULTRs were aligned using ClustalW v2.0 [23]. The neighbor-joining (NJ) method was used to construct the phylogenetic tree of the Gramineae species with 1000 bootstrap replicates [24]. The identified SULTRs were renamed according to the chromosomal location order in the genomes of each species.

### 2.2. Physical and Chemical Analysis of the SULTRs

The characteristics of SULTR, including the grand average of hydropathicity (GRAVY), molecular weight (MW) and isoelectric point (PI) were analyzed by the Sequence Manipulation Suite (http://www.detaibio.com/sms2/index.html, accessed on 20 March 2021). The subcellular localization of the SULTR proteins was detected by ProtComp 9.0 server (http://www.softberry.com/berry.phtml?topic=protcomp&group=help&subgroup=proloc, accessed on 20 March 2021) [25].

### 2.3. Gene Structure and Conserved Motif Analysis

The feature coordinates (exon–intron boundaries) were extracted from the GFF3 annotation files. The resulting BED format files were used as input for constructing the exon–intron structure of SULTRs in Gene Structure Display Server 2.0 (http://gsds.gao-lab.org/, accessed on 20 March 2021) [26]. SULTR motifs were investigated in the MEME suite (http://meme.suite.org/, accessed on 20 March 2021) with the number of motifs parameter set to 15 [27]. The combination of phylogenetic gene structure and motif information was performed by TBtools [28].

### 2.4. Gene Duplication Events, Chromosomal Locations and Orthogroup Analysis

Gene duplication of the SULTR family in each species was identified using the ‘duplicate_gene_classifier’ script in the Multiple Collinearity Scan Toolkit (MCScanX) with an e-value of 1 × 10^−^^10^ [29]. Comparative genome analysis was performed by using the MCScanX toolkit. The synteny analysis of the SULTR family in the 10 genomes was also conducted. All 10 representative genome annotation files were downloaded from Ensembl Plants (https://plants.ensembl.org/index.html, accessed on 20 March 2021). The chromosomal location of all the SULTRs was determined based on the genome annotation GFF3 files (https://plants.ensembl.org/index.html, accessed on 20 March 2021).

The orthogroup was identified using OrthoFinder v2 with a cut-off e-value of 1 × 10^−^^3^ [30]. STAG and STRID algorithms were used to rebuild the phylogenetic tree of the selected species based on the detected orthogroup. DnaSP 6.0 was used to generate the value of Tajima’s D in each orthogroup [31].

All cDNA and protein sequences were aligned by the “ParaAT.pl” script in ParaAT2.0 software with muscle blast analysis [32]. The nonsynonymous substitution ratios (Ka) and synonymous substitution ratios (Ks) of the duplicated gene pairs. together with their ratio. were analyzed using KaKs_Calculator2.0 software [33]. To investigate the divergence time between each duplicated gene pair, the genetic distances were calculated and compared based on the amino acid sequences using MEGA 7.0. Moreover, the selective forces on the orthogroups of SULTRs were calculated by Tajima’s D value with Dnasp 6.0 [29,34].

The synteny relations of *B. distachyon*, *H. vulgare*, *O. sativa* ssp. indica, *O. sativa* ssp. japonica, *O. barthii*, *O. glabbermia*, *O. rufipogon*, *S. bicolor*, *S. italica* and *Z. mays* were analyzed by using the Multiple Collinearity Scan toolkit X version (MCScan X) [29]. Then, the micro-syntenic pairs of the SULTRs family among the 10 genomes were constructed by TBtools.

### 2.5. Expression Analysis of SULTR Members in O. sativa ssp. indica

A total of 175 RNA-Seq datasets of different tissues at different developmental stages were downloaded from NCBI sequence read archive (SRA) (Supplementary Table S1). For RNA-Seq analysis, rice genome sequences and annotated gene files were downloaded from MBKbase (http://www.mbkbase.org/rice, accessed on 20 March 2021). Sequencing reads were aligned to R498 genome with TopHat version 2.0.13 [35]. The fragments per kilobase of exon model per million mapped reads (FKPM) method was used to estimate the gene expression using Cufflinks version 2.2.1 [36]. The detailed SRA information is described in the Supplementary Table S1. The average FKPM from the replicates was used in this study. Based on the clustering, the log_2_ values of the gene expression levels were used to create a heatmap, representing the relative expression of the 12 SULTR genes at different developmental stages.

### 2.6. Plant Material and Treatments

In this study, the elite restorer line (*Oryza sativa* ssp. indica, 9311) of HL-CMS was used to obtain the expression profiles of the SULTR genes under heat, drought, salt, low-selenium and high-selenium treatments at different time points. All seedlings were grown in a hydroponic box filled with Yoshida solution in a growth chamber at Wuhan University with the climatic conditions set at 28 °C day/18 °C night, 14 h light/10 h dark and 65% relative humidity for 12 days. For heat treatment, 12-day-old seedlings grown in Yoshida solution were transferred to a light incubator (Bluepard, Shanghai Bluepard Instruments Co., Ltd., Shanghai, China) with the temperature set to 40 °C (14 h/day, 10 h/night). Roots were collected at 0, 3, 6, 9, 12 and 24 h after the application of heat treatments. A 20% w/v polyethylene glycol (PEG-6000) solution was applied to imitate drought stress [37]. For the salt stress, low-selenium and high-selenium treatments, the roots of each seedling were washed, followed by immediate transfer into a 200 mM NaCl solution, 2 μM, and 20 μM Na_2_SeO_4_ solution, respectively [37,38]. Roots were sampled at 0, 3, 6, 9, 12 and 24 h during the light period after the application of treatments. Each treatment was conducted with 3 replicates in a completely randomized design. Three leaves at 0, 12, 24, 48 and 72 h after each treatment were randomly sampled and the fresh weight (FW) was immediately recorded. The leaf samples were then kept in a 50 mL tube filled with water for 12 h to obtain the turgid weight (TW) according to a previous report [39]. The the samples were then oven-dried at 80 °C for 48 h. The dry weight (DW) was recorded, and the relative water content was calculated according to the following expression: RWC = (FW − DW)/(TW − DW) × 100%. Student’s *t*-test was used to determine if the mean of RWC at different times under stress was significantly different from in normal conditions.

### 2.7. RNA Isolation and RT-PCR Analysis of OsiSULTRs under Stress

To investigate the function of the 12 *OsiSULTRs* in the rice cultivar 9311, the expression patterns were analyzed in shoots and roots under various stresses using qRT-PCR. Total RNA was extracted using TRIzol reagent (Invitrogen). A total of 20 μg RNA was digested with RNA-free DNase I (New England Biolabs) and then reverse-transcribed to cDNA using random primers. The quantitative real-time PCR (qRT-PCR) was performed using 2× SYBR Green Master Mix reagent (Roche). The *β-actin* gene served as an internal control and the primer sequences of *OsiSULTRs* were listed in Supplementary Table S2. Each sample was tested for 3 technical and 3 biological replicates.

## 3. Results

### 3.1. Identification and Comparative Phylogeny of SULTRs in Ten Gramineae Species

**Figure 1:**
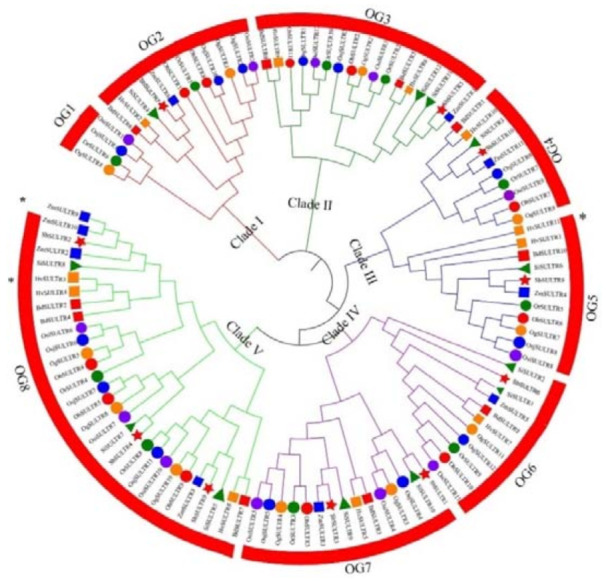
A NJ phylogeny tree of 10 Gramineae species’ SULTR protein sequences. Different colored branches represent different clades. Different shapes combined with different colors indicate the SULTRs in different species. Brown filled circles represent *Oryza glabbermia*. Green filled circles show *Oryza rufipogon*. Blue filled circles mean subspecies *Oryza sativa* ssp. japonica. Purple filled circles stand for *Oryza sativa* ssp. indica. Red squares denote *Brachypodium distachyon*. Brown squares stand for *Hordeum vulgare*. Green squares represent *Setaria italica*. Red squares mean *Sorghum bicolor*. Blue squares indicate *Zea mays*. Red filled circles denote *Oryza barthii.* The numbers outside the circles with red color represent the different orthogroups (OGs). * means proteins that are unassigned.

We identified 11, 10, 11, 11, 10, 12, 10, 11, 13 and 12 SULTRs in *O. barthii* (Ob), *B. distachyon* (Bd), *O. glabbermia* (Og), *H. vulgare* (Hv), *O. rufipogon* (Or), *S. italica* (Si), *S. bicolor* (Sb), *Z. mays* (Zm), *O. sativa* ssp. japonica (Osj, Nipponbare) and *O. sativa* ssp. indica (Osi, R498), respectively. The physiological and biochemical parameters are summarized in Supplementary Table S3. To understand the phylogenetic relationships of the SULTRs in the 10 Gramineae species, a phylogenetic tree was constructed and classification of the identified SULTRs was performed (Figure 1). Clade IV and Clade V had significant gene expansion compared with Clade I and Clade II. Clade I had a larger number of SULTRs than Clade II for *Oryza sativa* ssp. japonica (Supplementary Table S4). Osj, Osi and Si had a higher number of SULTRs than Hv, Bd, Zm, Ob, Og and Or. We found two distinct expansion patterns during Asian cultivated rice and African cultivated rice domestication. The results showed an expansion of SULTRs during the domestication of Asian cultivated rice, while a loss of SULTRs occurred during the domestication of the African cultivated rice.

To better understand the evolutionary pattern of SULTRs in Gramineae, the orthogroup (OG) clustering of the identified SULTRs was determined. A total of 111 SULTRs were classified into eight OGs, except for three unassigned SULTRs (Figure 1, Table 1). We found that the number of genes in each OG was different, with a range from 4 to 27. Of the classified OGs, the 10 species shared seven OGs (OG2–OG8), and single-copy gene clusters were found in OG4 and OG5. OG1 exists in all rice species except for *Oryza barthii*. Taking all results into account, we discovered that unequal loss and expansion of most OGs occurred during the domestication process, except for the conserved OG4 and OG5. In addition, the Tajima’s D values of all OGs were less than 0, indicating that the SULTRs in the different OGs were under strong negative selection (Table 1).

### 3.2. Expansion Patterns and Chromosome Location of SULTRs in 10 Gramineae Species

**Figure 2:**
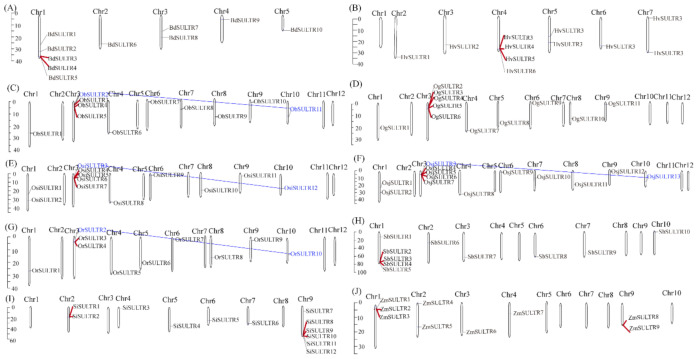
The chromosome location and duplication events of SULTR genes in 10 grass plants, namely *B. distachyon* (**A**), *H. vulgare* (**B**), *O. barthii* (**C**), *O. glabberrima* (**D**), *O. sativa* ssp. indica (**E**), *O. sativa* ssp. japonica (**F**), *O. rufipogon* (**G**), *S. italica* (**H**), *S. bicolor* (**I**) and *Z. mays* (**J**). The red lines indicate tandem duplications and the blue lines with blue font labels show whole-genome duplications (WGD)/segmental duplication events.

To understand the SULTRs’ expansion mechanism among the 10 Gramineae species, we investigated the chromosomal distribution and duplication types of SULTRs within each species. The SULTRs of different Gramineae species showed various distributions and densities on chromosomes. According to Figure 2, a total of 25 duplicated gene pairs were identified in the 10 representative species (Table 2), including two duplicate modes: whole-genome duplication (WGD)/segmental duplication and tandem duplication. WGD/segmental duplications were only observed in rice species, including *Oryza barthii*, *Oryza sativa* ssp. indica, *Orya sativa* ssp. japonica and *Oryza rufipogon*. The duplicated gene pairs in the SULTRs accounted for 10 to 31% among the 10 Gramineae genomes. The duplicate modes and the numbers of SULTRs varied among the 10 genomes, especially in rice species. Although the total number of duplicate gene pairs was constant during the domestication from *Oryza barthii* to *Oryza glabbermia*, one WGD/segmental duplication and two tandem duplications were found in *Oryza barthii* and only tandem duplications were found in *Oryza glabbermia*. During the domestication of Asian cultivated rice, the numbers and types of duplicated gene pairs were consistent between Osi and Or, while Osj showed two more tandem duplicated gene pairs compared with Or.

**Figure 3:**
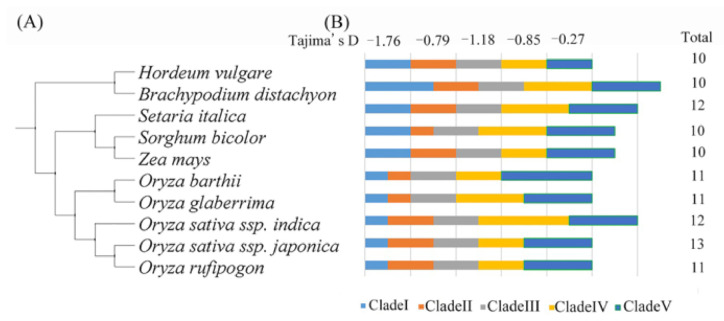
The SULTR numbers and Tajima’s D values of the five clades among 10 grass plants. (**A**) The phylogenetic tree of the 10 species was generated based on the orthogroups results from OrthoFinder software. (**B**) Histogram charts of different subfamilies in *H. vulgare*, *B. distachyon*, *O. barthii, O. glabbermia, O. sativa* ssp. indica, *O. sativa* ssp. *japonica*, *O. rufipogon*, *S. italica*, *S. bicolor* and *Z. mays*. The total orthogroups numbers of SULTRs in these 10 plants are shown on the right side.

The divergence times of all duplicated gene pairs ranged from 5.95 to 168.30 Mya and the segmental duplications of SULTR genes pairs occurred from 32.92 to 80.72 Mya. These results showed the high variation of the divergence time among the 10 species. For example, the divergence time of duplicated gene pairs in Bd was 168.11 Mya; it ranged from 10.06 to 151.07 Mya in Hv and from 136.63 to 151.99 Mya in Sb. The Ka/Ks values ranged from 0.07 to 0.90, indicating that all duplicated gene pairs were under strong purifying selection during Gramineae’s evolution (Table 1). In addition, the Tajima’s D values of each clade were less than 0, suggesting that the SULTR genes in each clade were under purifying selection (Figure 3).

### 3.3. Gene Structure and Motif Patterns of SULTRs

**Figure 4:**
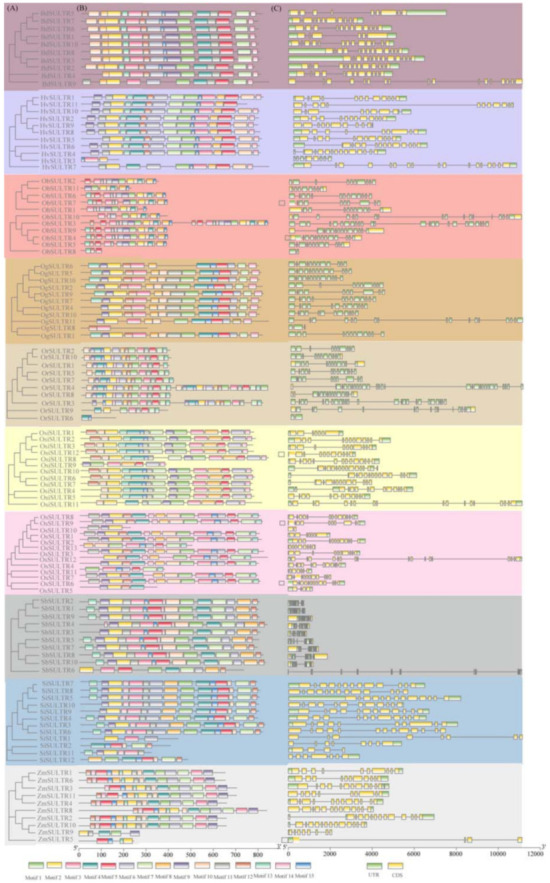
Structure of SULTR genes and proteins. Different colors represent the different Gramineae species. (**A**) A phylogenetic tree of SULTR protein sequences from the 10 Gramineae species. (**B**) Distribution of the conserved motifs of the SULTRs in the 10 Gramineae species. (**C**) Exon–intron structures of the SULTRs in the 10 selected plants.

The numbers of introns in the SULTRs ranged from 6 to 17, and no significant features were found between different clusters in each species (Figure 4). A total of 15 conserved motifs were identified and assigned to Motifs 1 to 15. Nine of them were found to be highly conserved. The sequences and conserved motifs are listed in Supplementary Tables S5 and S6. Genes that shared motif patterns were clustered into one group, indicating a similar function. These results suggest that the diverse motif composition among the SULTRs may have different functions in Gramineae species.

### 3.4. Syntenic Relationship of SULTRs among the Selected Species

**Figure 5:**
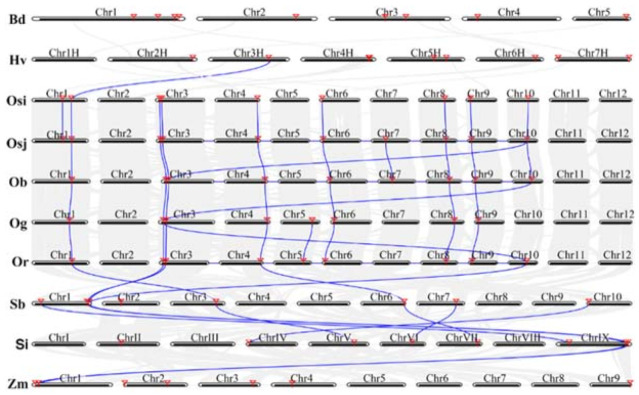
Syntenic relationships of SULTR genes among the 10 Gramineae species. The gray lines indicate the syntenic genes between two species, the blue lines show the syntenic SULTR genes between two species and the red triangles stand for the locations of the SULTR genes in each genome.

The misidentification of highly conserved paralogs could result from the rapid divergence of the orthologous genes after speciation or gene loss after duplication [40]. To further elucidate the SULTR gene family size variation within the Gramineae species, the synteny of the SULTRs was investigated by comparative genome analysis of the 10 selected species using the MCScanX toolkit. We observed that the number of syntenic gene pairs among Bd, Hv, Osi, Osj, Ob, Og, Sb, Si and Zm ranged from 0 to 11 (Figure 5). The syntenic relationship of the SULTRs was highly conserved among rice species. However, we identified only a weak syntenic relationship between Hv and Osi and between Zm and Si. Among them, Bd and Hv exhibited the lowest syntenic relationship of SULTRs, which indicateed that Bd has a distant relationship with Hv. These findings reveal closer relationships in rice species compared with other selected Gramineae species, consistent with their evolutionary distance.

### 3.5. Constitutive Expression Pattern of SULTRs in Oryza sativa ssp. indica

**Figure 6:**
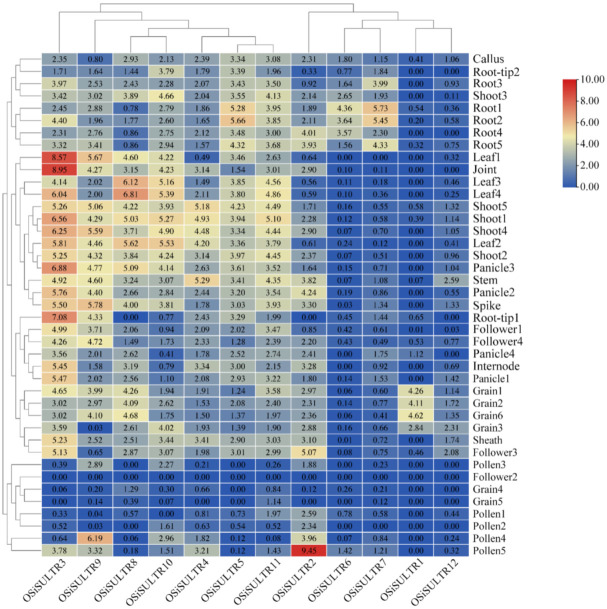
Expression patterns of *OsiSULTR* genes in different tissues at different stages. The values in the heatmap calculated by log_2_ FPKM represent the relative expression of *OsiSULTRs* derived from the public datasets. Green/red color indicates the high/low expression level of transcripts. The IDs corresponding to the different tissues at different stages are listed in Supplementary Table S1.

To assess the function of the identified SULTRs in *Oryza sativa* ssp. indica (R498), we analyzed public rice transcriptomic data from all tissues at different stages. The results showed that the 12 *OsiSULTRs* exhibited distinct spatial and temporal expression patterns and can be divided into five groups (Figure 6). The first *OsiSULTR* group was only expressed in grain and had almost no expression in other tissues (for example, *OsiSULTR1*). The second group had notable expression levels in leaves, with relatively low expression in other tissues at all stages (for example, *OsiSULTR3* and *OsiSULTR8*). The third group (*OsiSULTR6* and *OsiSULTR7*) was only expressed in the roots and had low expression levels in other tissues at all stages. The fourth group (*OsiSULTR2*) was strongly expressed in pollen and showed no expression in most tissues at all stages. The last group was expressed in all tissues at least at one stage. All *OsiSULTRs* were expressed in the roots except *OsiSULTR1*, while two *OsiSULTRs* (*OsiSULTR1* and *OsiSULTR6*) had no expression in the shoots of 14-day-old seedlings. These results imply that *OsiSULTRs* is expressed in a tissue-specific manner in rice.

### 3.6. Expression Profiling of SULTRs in Rice Roots under Stress Conditions

We further investigated the SULTRs’ expression under different abiotic stresses, including salt, heat, low-selenium, high-selenium and drought stress. First, the relative water content (RWC) of seedlings under each stress was evaluated (Supplementary Figure S1). Among the stresses, the RWC values under low-selenium and normal conditions showed no significant difference, while RWC values under the remaining stresses and in the control showed significant differences. The results indicated that low-selenium stress may have no effect on the rice seedling, while other abiotic stresses have significant impact on rice (Supplementary Figure S1).

**Figure 7:**
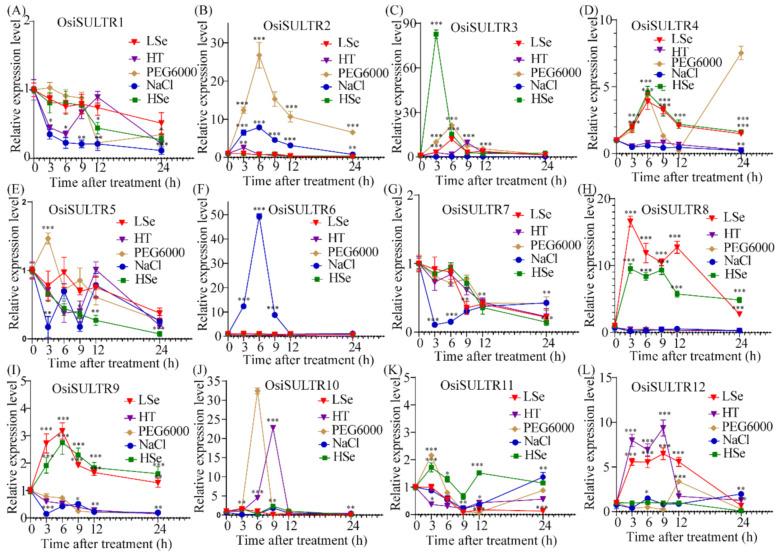
Relative expression of *OsiSULTR* in roots at the rice seedling stage under salt stress, high-temperature stress and drought stress by qRT-PCR. (**A**–**L**) The expression profiles of 12 OsiSULTRs in rice cultivar 9311 by qRT-PCR. LSe, HSe, PEG6000, HT, and NaCl represent the low-selenium treatment, the high-selenium treatment, polyethylene glycol treatment, high-temperature stress and salt stress. Student’s *t*-test was used in this experiment; *: *p* < 0.05, **: *p* < 0.05, ***: *p* < 0.001.

Next, the expression patterns of *OsiSULTRs* in roots were measured by qRT-PCR (Figure 7). *OsiSULTR2* and *OsiSULTR6* were significantly upregulated at almost all time points under salt stress, while the expression levels of *OsiSULTR10* and *OsiSULTR12* were elevated at 6 and 9 h after salt treatment, respectively. The expression levels of two SULTR genes (*OsiSULTR3* and *OsiSULTR10*) were increased after the heat and drought treatments. Furthermore, *OsiSULTR12* and *OsiSULTR2* were upregulated under heat and PEG treatments, respectively. Taking together, *OsiSULTR2* showed significant upregulation under salt and drought stress, combined with one time point of heat stress. *OsiSULT3*, *OsiSULTR4*, *OsiSULTR8* and *OsiSULTR9* were upregulated under both low-selenium and high-selenium stress. *OsiSULTR10* exhibited upregulation under the low-selenium treatment and downregulation under the high-selenium treatment. *OsiSULTR11* and *OsiSULTR12* showed different expression patterns under the selenium treatments.

### 3.7. Expression Profiling of SULTRs in Rice Shoots under Stress Condition

**Figure 8:**
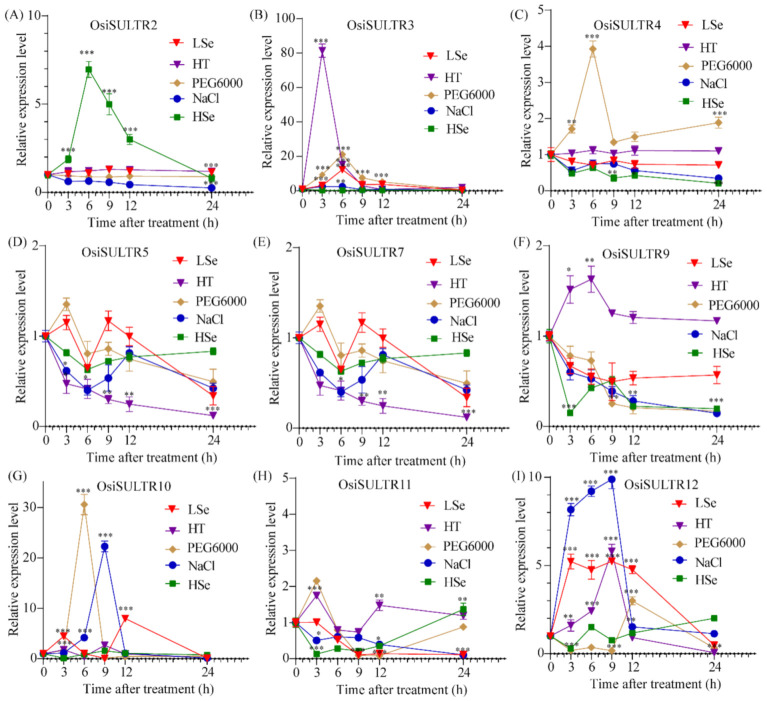
Expression patterns of *OsiSULTRs* in shoots under five abiotic stress in rice at the seedling stage. (**A**–**I**) The expression profiles of nine *OsiSULTRs* in rice cultivar 9311 by qRT-PCR. LSe, HSe, PEG6000, HT and NaCl indicate the low-selenium treatment, the high-selenium treatment, the polyethylene glycol treatment, the high-temperature treatment and salt stress. Student’s *t*-test was used in this experiment; *: *p* < 0.05, **: *p* < 0.05, ***: *p* < 0.001.

Previous studies demonstrated that *OsiSULTR* transcript levels in shoots were post-transcriptionally modulated in response to changes in S conditions. Hence, we evaluated the *OsiSULTR* expression patterns in shoots under the salt, high temperature, drought, high-selenium and low-selenium treatments (Figure 8). The results showed that *OsiSULTR2* was upregulated by the high-selenium treatment at all the tested time points and the expression was highest at 6 h after treatment. *OsiSULTR3, OsiSULTR10* and *OsiSULTR12* showed significantly higher expression levels than other *OsiSULTRs* under the low-selenium treatment. The expression levels of *OsiSULTR10* and *OsiSULTR12* were significantly upregulated under salinity stress. Under the PEG6000 treatment, the expression levels of *OsiSULTR3, OsiSULTR4, OsiSULTR10, OsiSULTR11* and *OsiSULTR12* were increased compared with the control. *OsiSULTR3, OsiSULTR9* and *OsiSULTR12* were strongly induced in shoots by the high-temperature stress compared with the control. In short, *OsiSULTR3* positively responded to the low-selenium, high-selenium, high-temperature and drought stresses, while *OsiSULTR12* was upregulated under all stresses except for the high-selenium treatment. Furthermore, *OsiSULTR1*, *OsiSULTR6* and *OsiSULTR8* were not expressed in the shoots of 12-day-old seedlings under the salt, high-temperature, drought, high-selenium and low selenium treatments.

## 4. Discussion

High-quality grass family (Gramineae) genome sequences have brought opportunities to investigate the short-term evolutionary dynamics of the SULTR family. The synteny of their genomes also facilitated the comparative analysis of SULTR genes’ structure and function. In our study, 111 SULTRs from 10 Gramineae species were identified. The results revealed no direct correlation between the number of SULTR genes and genome size. For instance, there were 12 SULTR genes in *S. bicolor* (genome size: 730 Mbp) [41], while there were 10 SULTR genes in *H. vulgare* (genome size: 4.79 Gbp) [42]. *Zea mays*, which experienced a specific WGD absent in other Gramineae crops [43], has the fewest SULTRs among the 10 Gramineae species in our study. This result indicates that *Zea mays* lost SULTRs after the gene duplication events. In the orthogroup clustering, *Zea mays* also showed fewer SULTRs than the remaining species in OG2 and OG4 (Table 2). Previous studies reported that tandem duplication and WGD/segmental duplication played a critical role in gene family expansion [44]. We found that the expansion mechanisms of the SULTRs family were different among the Gramineae species. For instance, no WGDs/segmental duplication events occurred in the studied Gramineae species apart from the rice species. SULTRs in Si had syntenic relationships with most of the SULTRs in other grass crops except *Oryza*. These results indicate that the expansion of the SULTR family in Si might have resulted from other unknown duplication types or large rearrangements, such as dispersed, proximal, inversions and translocations.

The number of SULTRs detected in this study is consistent with previous studies [15,16]. However, two sulfate transporters, *OsSultr1*;*3* (*LOC_os08g031410*) and *OsSultr5*;*1* (*LOC_os08g031410*), were not identified in this study. Instead, we identified one new SULTR gene (*LOC_Os07g18700*) in *Oryza sativa* ssp. japonica. Different gene structural arrangements and diverse expression patterns led to different functions of the gene subfamilies. Thus, we classified the 111 SULTR proteins into five clades. Clades I to III were classified into Group 3, whose functions have not been clearly investigated to date. Clade IV was equivalent to Group 2, corresponding to low-affinity transporters, and Clade V was classified as Group 1, involving high-affinity transporters. The gene expression profiling of the SULTRs also supported the classification of SULTRs based on Figure 6. Moreover, the SULTRs showed different gene structures between different clades, which confirmed the clade classification (Figure 4). The Tajima’s D values of all OGs in the five clades and the Ka/Ks values of the 25 duplicated gene pairs indicated that the SULTR family in the 10 Gramineae plants were under strong purifying selective forces. Additionally, with a total of eight OGs identified, OG8 (Group 3) contained 27 SULTRs, while OG1 (one from Group 3) had only four SULTRs. This suggested that different OGs might suffer from various extents of gene expansion and loss. We hypothesized that the various numbers of SULTRs identified in the OGs or clades was a result of natural or artificial selection. Multiple copies of high-affinity and low-affinity transporters were identified, while transporters with unclear functions were lost during rice domestication, according to the comparisons between Ob and Oj, Or and Osj, and Or and Osi.

Until now, increasing evidence has reported that SULTRs are critical for plant sulfate uptake and transport [45]. Our results showed that SULTRs have different expression patterns in different tissues, and some SULTRs had tissue-specific expression. We found that high-affinity SULTR genes showed increased expression in roots, which supported the idea that SULTRs participate in the uptake of sulfate from the soil and the source–sink transport of sulfate [38]. In addition, low-affinity transporter SULTR genes had high expression levels in the stem, which suggests that the SULTRs in Group 2 are involved in regulating vascular sulfate transport. In addition, the other SULTRs did not show tissue-specific expression patterns but were expressed in most tissues during the developmental stages. Previous studies reported that SULTRs have positive responses to plant abiotic stress [11,46]. In our study, we identified 12 *OsiSULTRs* in shoots and roots that respond to salinity, high temperature, drought, high-selenium and low-selenium treatments. The results showed that three *OsiSULTRs* had no expression under five stresses in the shoots, while all *OsiSULTRs* were expressed in the roots. *OsiSULTR1* and *OsiSULTR6* were not expressed in the shoots either in constitutive expression patterns based on resequencing data or under abiotic stress detected by qRT-PCR. The expression of *OsiSULTR8* has not been detected, while *OsiSULTR2* was expressed in shoots, detected by qRT-PCR.

*OsiSULTR10* was significantly upregulated in both shoots and roots under salinity stress. Meanwhile, *OsiSULTR3* was remarkably upregulated under salt stress, high temperature and PEG6000. These results imply that *OsSULTRs* play different roles in low-selenium, high-temperature, drought and salt stress tolerance. Using *Oryza sativa* as a model species, the expression and clustering results of SULTRs in this study shed light on future functional analysis in Gramineae species.

## 5. Conclusions

In this study, 111 SULTRs were identified in Gramineae genomes, including 11, 10, 12, 11, 13, 10, 12, 11, 12 and 10 SULTRs in *B. distachyon*, *H. vulgare*, *O. barthii*, *O. glabbermia*, *O. sativa* ssp. japonica, *O. sativa* ssp. indica, *O. rufipogon*, *S. bicolor*, *S. italica* and *Z. mays*, respectively. Our results revealed the different expansion mechanisms of the SULTR family among the 10 selected Gramineae species and detected various selective pressures in the clustered clades and orthogroups. The SULTRs highly expressed in the shoots and roots may be essential for sulfur uptake and transport. The qRT-PCR results suggested that SULTR genes have important functions in response to salt, heat, drought, low-selenium and high-selenium stresses. Taken together, our results will facilitate further studies of SULTR genes’ functions in other Gramineae plants.

## Figures and Tables

**Table 1:** Orthologous groups numbers and genetic diversity in 10 Gramineae species.

	Bd	Hv	Ob	Og	Osj	Osi	Or	Sb	Si	Zm	Tajima’s D
OG1	0	0	0	1	1	1	1	0	0	0	−1.4196
OG2	1	1	2	1	2	1	1	1	1	1	−1.5876
OG3	2	2	2	2	2	2	1	1	2	1	−0.7869
OG4	1	1	1	1	1	1	1	1	1	1	−1.3148
OG5	1	1	1	1	1	1	1	1	1	1	−1.5443
OG6	1	1	1	1	1	1	1	1	2	1	−1.4481
OG7	1	1	1	2	2	2	1	2	2	1	−1.1917
OG8	3	2	3	3	3	3	2	2	2	2	−0.2679

**Table 2:** Synonymous/nonsynonymous mutations and the divergence time of duplicated gene pairs in 10 selected genomes.

Seq_A	Seq_B	Ka	Ks	Ka/Ks	Duplication Mode	Time (MY)	Purifying Selection
BdSULTR3	BdSULTR4	0.4762	2.5145	0.189384	Tandem duplication	138.15	Yes
HvSULTR3	HvSULTR4	0.0411	0.1831	0.224279	Tandem duplication	10.06	Yes
HvSULTR4	HvSULTR5	0.4533	2.7496	0.164851	Tandem duplication	151.07	Yes
ObSULTR3	ObSULTR4	0.4364	2.5439	0.1716	Tandem duplication	139.77	Yes
ObSULTR4	ObSULTR5	0.1529	0.5991	0.2553	Tandem duplication	32.92	Yes
ObSULTR3	ObSULTR11	0.1707	1.4530	0.1175	WGD/segmental duplication	80.72	Yes
OgSULTR3	OgSULTR4	0.2169	3.0507	0.0711	Tandem duplication	167.62	Yes
OgSULTR4	OgSULTR5	0.4233	2.7101	0.1562	Tandem duplication	148.9	Yes
OgSULTR5	OgSULTR6	0.2578	0.6580	0.3918	Tandem duplication	36.15	Yes
OsiSULTR4	OsiSULTR5	0.2184	3.0630	0.0713	Tandem duplication	168.30	Yes
OsiSULTR6	OsiSULTR7	0.1275	0.5593	0.2280	Tandem duplication	30.73	Yes
OsiSULTR3	OsiSULTR12	0.1745	1.3397	0.1302	WGD/segmental duplication	74.43	Yes
OsjSULTR4	OsjSULTR5	0.2951	2.8665	0.1029	Tandem duplication	157.5	Yes
OsjSULTR5	OsjSULTR6	0.4427	2.9321	0.1510	Tandem duplication	161.10	Yes
OsjSULTR6	OsjSULTR7	0.1530	0.6024	0.2540	Tandem duplication	33.1	Yes
OsjSULTR3	OsjSULTR13	0.1755	1.8594	0.0944	WGD/segmental duplication	102.16	Yes
OrSULTR3	OrSULTR4	0.4487	2.5274	0.1775	Tandem duplication	138.87	Yes
OrSULTR2	OrSULTR10	0.1726	1.2921	0.1335	WGD/segmental duplication	70.99	Yes
SbSULTR2	SbSULTR3	0.4604	2.4867	0.1852	Tandem duplication	136.63	Yes
SbSULTR3	SbSULTR4	0.2288	3.0597	0.0748	Tandem duplication	168.11	Yes
SiSULTR1	SiSULTR2	0.9085	1.2309	0.7380	Tandem duplication	67.63	Yes
SiSULTR8	SiSULTR9	0.4499	2.4567	0.1831	Tandem duplication	134.98	Yes
SiSULTR9	SiSULTR10	0.2022	2.7663	0.0731	Tandem duplication	151.99	Yes
ZmSULT2	ZmSULT3	0.4496	2.5814	0.1741	Tandem duplication	141.84	Yes
ZmSULT9	ZmSULT10	0.0972	0.1082	0.8986	Tandem duplication	5.94	Yes

## Data Availability

Data is contained in supplementary material.

## Supplementary Materials

The following are available online at https://www.mdpi.com/article/10.3390/genes12050634/s1, Table S1: RNA-Seq datasets for build expression atlas, Table S2: List of oligonucleotide primary used for qRT-PCR, Table S3: The physiological and phylogenetic relationships of the SULTRs in Gramineae crops, Table S4: Gramineae SULTR gene numbers in different clades, Table S5: The protein sequences of rice SULTRs, Table S6: The conserved motifs of rice SULTRs, Figure S1: The relative water content of rice at different time points under different stresses.

## References

[1] Buchner P., Parmar S., Kriegel A., Carpentier M., Hawkesford M.J. (2010). The sulfate transporter family in wheat: Tissue-specific gene expression in relation to nutrition. Mol. Plant.

[2] Hart J.W., Filner P. (1969). Regulation of sulfate uptake by amino acids in cultured tobacco cells. Plant Physiol..

[3] Saito K. (2000). Regulation of sulfate transport and synthesis of sulfur-containing amino acids. Curr. Opin. Plant Biol..

[4] Furner I.J., Sung Z.R. (1982). Regulation of sulfate uptake in carrot cells: Properties of a hypercontrolled variant. Proc. Natl. Acad. Sci. USA.

[5] Smith F.W., Ealing P.M., Hawkesford M.J., Clarkson D.T. (1995). Plant members of a family of sulfate transporters reveal functional subtypes. Proc. Natl. Acad. Sci. USA.

[6] Takahashi H. (2010). Regulation of sulfate transport and assimilation in plants. Int. Rev. Cell Mol. Biol..

[7] El-Soda M., Kruijer W., Malosetti M., Koornneef M., Aarts M.G. (2015). Quantitative trait loci and candidate genes underlying genotype by environment interaction in the response of Arabidopsis thaliana to drought. Plant Cell Environ..

[8] Geng Y., Wu R., Wee C.W., Xie F., Wei X., Chan P.M., Tham C., Duan L., Dinneny J.R. (2013). A spatio-temporal understanding of growth regulation during the salt stress response in Arabidopsis. Plant Cell.

[9] Takahashi H. (2003). Functions of sulfate transporters in plants. Plant Cell Physiol..

[10] Sacchi G.A., Nocito F.F. (2019). Plant sulfate transporters in the low phytic acid network: Some educated guesses. Plants.

[11] Huang Q., Wang M.P., Xia Z.L. (2018). The SULTR gene family in maize (*Zea mays* L.): Gene cloning and expression analyses under sulfate starvation and abiotic stress. J. Plant Physiol..

[12] Gigolashvili T., Kopriva S. (2014). Transporters in plant sulfur metabolism. Front. Plant Sci..

[13] Ding Y., Zhou X., Zuo L., Wang H., Yu D. (2016). Identification and functional characterization of the sulfate transporter gene GmSULTR1; 2b in soybean. BMC Genom..

[14] Buchner P., Stuiver C.E., Westerman S., Wirtz M., Hell R., Hawkesford M.J., De Kok L.J. (2004). Regulation of sulfate uptake and expression of sulfate transporter genes in Brassica oleracea as affected by atmospheric H(2)S and pedospheric sulfate nutrition. Plant Physiol..

[15] Kumar S., Asif M.H., Chakrabarty D., Tripathi R.D., Trivedi P.K. (2011). Differential expression and alternative splicing of rice sulphate transporter family members regulate sulphur status during plant growth, development and stress conditions. Funct. Integr. Genom..

[16] Buchner P., Takahashi H., Hawkesford M.J. (2004). Plant sulphate transporters: Co-ordination of uptake, intracellular and long-distance transport. J. Exp. Bot..

[17] Zhang J., Zhao C.Y., Liu J., Song R., Du Y.X., Li J.Z., Sun H.Z., Duan G.L., Zhao Q.Z. (2016). Influence of sulfur on transcription of genes involved in arsenic accumulation in rice grains. Plant Mol. Biol. Rep..

[18] Zhao H., Frank T., Tan Y., Zhou C., Jabnoune M., Arpat A.B., Cui H., Huang J., He Z., Poirier Y. (2016). Disruption of OsSULTR3;3 reduces phytate and phosphorus concentrations and alters the metabolite profile in rice grains. New Phytol..

[19] Mameaux S., Cockram J., Thiel T., Steuernagel B., Stein N., Taudien S., Jack P., Werner P., Gray J.C., Greenland A.J. (2012). Molecular, phylogenetic and comparative genomic analysis of the cytokinin oxidase/dehydrogenase gene family in the Poaceae. Plant Biotechnol. J..

[20] Silva C., Snak C., Schnadelbach A.S., van den Berg C., Oliveira R.P. (2016). Phylogenetic relationships of Echinolaena and Ichnanthus within Panicoideae (Poaceae) reveal two new genera of tropical grasses. Mol. Phylogen. Evol..

[21] Ye J., McGinnis S., Madden T.L. (2006). BLAST: Improvements for better sequence analysis. Nucleic. Acids. Res..

[22] Marchler-Bauer A., Lu S., Anderson J.B., Chitsaz F., Derbyshire M.K., DeWeese-Scott C., Fong J.H., Geer L.Y., Geer R.C., Gonzales N.R. (2011). CDD: A Conserved Domain Database for the functional annotation of proteins. Nucleic. Acids. Res..

[23] Chenna R., Sugawara H., Koike T., Lopez R., Gibson T.J., Higgins D.G., Thompson J.D. (2003). Multiple sequence alignment with the Clustal series of programs. Nucleic. Acids. Res..

[24] Kumar S., Stecher G., Tamura K. (2016). MEGA7: Molecular evolutionary genetics analysis version 7.0 for bigger datasets. Mol. Biol. Evol..

[25] Klee E.W., Ellis L.B. (2005). Evaluating eukaryotic secreted protein prediction. BMC Bioinform..

[26] Hu B., Jin J., Guo A.Y., Zhang H., Luo J., Gao G. (2015). GSDS 2.0: An upgraded gene feature visualization server. Bioinformatics.

[27] Bailey T.L., Johnson J., Grant C.E., Noble W.S. (2015). The MEME Suite. Nucleic. Acids. Res..

[28] Chen C., Chen H., Zhang Y., Thomas H.R., Frank M.H., He Y., Xia R. (2020). TBtools: An integrative toolkit developed for interactive analyses of big biological data. Mol. Plant.

[29] Wang Y., Tang H., Debarry J.D., Tan X., Li J., Wang X., Lee T.H., Jin H., Marler B., Guo H. (2012). MCScanX: A toolkit for detection and evolutionary analysis of gene synteny and collinearity. Nucleic. Acids. Res..

[30] Emms D.M., Kelly S. (2019). OrthoFinder: Phylogenetic orthology inference for comparative genomics. Genome. Biol..

[31] Rozas J., Ferrer-Mata A., Sanchez-DelBarrio J.C., Guirao-Rico S., Librado P., Ramos-Onsins S.E., Sanchez-Gracia A. (2017). DnaSP 6: DNA sequence polymorphism analysis of large data sets. Mol. Biol. Evol..

[32] Zhang Z., Xiao J., Wu J., Zhang H., Liu G., Wang X., Dai L. (2012). ParaAT: A parallel tool for constructing multiple protein-coding DNA alignments. Biochem. Biophys. Res. Commun..

[33] Wang D., Zhang Y., Zhang Z., Zhu J., Yu J. (2010). KaKs_Calculator 2.0: A toolkit incorporating γ-series methods and sliding window strategies. Genom. Proteom. Bioinform..

[34] Korneliussen T.S., Moltke I., Albrechtsen A., Nielsen R. (2013). Calculation of Tajima’s D and other neutrality test statistics from low depth next-generation sequencing data. BMC Bioinform..

[35] Trapnell C., Pachter L., Salzberg S.L. (2009). TopHat: Discovering splicing junctions with RNA-Seq. Bioinformatics.

[36] Trapnell C., Williams B.A., Pertea G., Mortazavi A., Kwan G., Van Baren M.J., Salzberg S.L., Wold B.J., Pachter L. (2010). Transcrit assembly and quantification by RNA-Seq reveals unannotated transcripts and isform switching during cell differentiation. Nat. Biotechnol..

[37] Shibagaki N., Rose A., McDermott J.P., Fujiwara T., Hayashi H., Yoneyama T., Davies J.P. (2002). Selenate-resistant mutants of Arabidopsis thaliana identify Sultr1;2, a sulfate transporter required for efficient transport of sulfate into roots. Plant J..

[38] Xiong H., Yu J., Miao J., Li J., Zhang H., Wang X., Liu P., Zhao Y., Jiang C., Yin Z. (2018). Natural variation in OsLG3 increases drought tolerance in rice by inducing ROS scavenging. Plant Physiol..

[39] Mostofa M.G., Fujita M. (2013). Salicylic acid alleviates copper toxicity in rice (*Oryza sativa* L.) seedlings by up-regulating antioxidative and glyoxalase systems. Ecotoxicology.

[40] Jacquemin J., Ammiraju J.S.S., Haberer G., Billheimer D.D., Yu Y., Liu L.N.C., Rivera L.F., Mayer K., Chen M.S., Wing R.A. (2014). Fifteen million years of evolution in the Oryza genus shows extensive gene family expansion. Mol. Plant.

[41] Paterson A.H., Bowers J.E., Bruggmann R., Dubchak I., Grimwood J., Gundlach H., Haberer G., Hellsten U., Mitros T., Poliakov A. (2009). The sorghum bicolor genome and the diversification of grasses. Nature.

[42] Beier S., Himmelbach A., Colmsee C., Zhang X.Q., Barrero R.A., Zhang Q., Li L., Bayer M., Bolser D., Taudien S. (2017). Construction of a map-based reference genome sequence for barley, *Hordeum vulgare* L. Sci. Data.

[43] Swigonova Z., Lai J., Ma J., Ramakrishna W., Llaca V., Bennetzen J.L., Messing J. (2004). Close split of sorghum and maize genome progenitors. Genome Res..

[44] Takahashi H., Kopriva S., Giordano M., Saito K., Hell R. (2011). Sulfur assimilation in photosynthetic organisms: Molecular functions and regulations of transporters and assimilatory enzymes. Annu. Rev. Plant Biol..

[45] Gabaldon T., Koonin E.V. (2013). Functional and evolutionary implications of gene orthology. Nat. Rev. Genet..

[46] Akbudak M.A., Filiz E., Kontbay K. (2018). Genome-wide identification and cadmium induced expression profiling of sulfate transporter (SULTR) genes in sorghum (Sorghum bicolor L.). Biometals.

