# Supplementary material for: Genome-Wide Identification and Expansion Patterns of SULTR Gene Family in Gramineae Crops and Their Expression Profiles under Abiotic Stress in Oryza sativa
Source: Genes (Basel). 2021 Apr 23;12(5):634. doi: 10.3390/genes12050634 (PMC8146379; doi:10.3390/genes12050634)
Supplement: Supplementary file 1 [file genes-12-00634-s001.zip › supplementary table 1.pdf]

Supplementary table 1. RNA-Seq Datasets of Rice For Build Expression Atlas

Tissue at different stages	Renamed tissue	NCBI Sequence Read Archive (SRA) ID	Instrument	Type of short read	Replicates	Download link
14-day Callus	Callus	SRR037713,SRR037712, SRR037711,SRR358797, SRR358796,SRR358795,	Illumina Genome Analyzer	Paired-end reads	Two biological replicates and three technical replicates	https://www.ncbi.nlm.nih.gov/bioproject/PRJNA117345
Roots at 0 day	Root1	DRR002726,DRR002725,DRR002724,DRR002713,DRR002712,DRR002711,DRR002710, DRR002699, DRR002698, DRR002697, DRR002696, DRR002685, DRR002684, DRR002683, DRR002682	Illumina Genome Analyzer IIx	Single-end reads	Five biological replicates and three technical replicates	https://www.ncbi.nlm.nih.gov/bioproject/PRJDB2658
Roots at 7 days	Root2	DRR001046, DRR001039, DRR001032, DRR001025	Illumina Genome Analyzer IIx	Single-end reads	Two biological replicates and three technical replicates	https://www.ncbi.nlm.nih.gov/bioproject/PRJDA67119

Root at 14 days	Root3	SRR037740, SRR306323, SRR306322	Illumina Genome Analyzer	Single-end reads	Three technical replicates	https://www.ncbi.nlm.nih.gov/bioproject/PRJNA117345
Root at 20 days	Root4	SRR306324,SRR306325	Illumina Genome Analyzer II	Paired-end reads	Two technical replicates	https://www.ncbi.nlm.nih.gov/bioproject/PRJNA143417
Root at 22 days	Root5	DRR002730, DRR002729, DRR002728, DRR002727	Illumina Genome Analyzer IIx	Single-end reads	Two biological replicates and two technical replicates	https://www.ncbi.nlm.nih.gov/bioproject/PRJDB2658
Root tips at 14 days	Root-tip1	SRR306318, SRR306317, SRR30316	Illumina Genome Analyzer II	Single-end reads	Three technical replicates	https://www.ncbi.nlm.nih.gov/bioproject/PRJNA143417
Root tips at 20 days	Root-tip2	SRR306321, SRR306320, SRR306319	Illumina Genome Analyzer II	Single-end reads	Three biological replicates	https://www.ncbi.nlm.nih.gov/bioproject/PRJNA143417
Shoot at 0 day	Shoot1	DRR002734, DRR002733, DRR002732, DRR002731, DRR002720, DRR002719, DRR002718, DRR002706, DRR002705, DRR002704, DRR002692, DRR002691, DRR002690	Illumina Genome	Single-end reads	Four biological replicates	https://www.ncbi.nlm.nih.gov/bioproject/PRJDB2658

			Analyzer IIx		and three technical replicates	
Shoot at 7 days	Shoot2	DRR002734, DRR001040, DRR001023, DRR001026	Illumina Genome Analyzer IIx	Single-end reads	Two biological replicates and three technical replicates	https://www.ncbi.nlm.nih.gov/bioproject/PRJDB2658
Shoot at 14 days	Shoot3	SRR037441, SRR2467253, SRR2467252	Illumina HiSeq 2000	Single-end reads	Three technical replicates	https://www.ncbi.nlm.nih.gov/bioproject/PRJNA79561
Shoot at 22 days	Shoot4	DRR002738, DRR002737, DRR002736, DRR002735, DRR002723, DRR002722, DRR002721, DRR002709, DRR002708, DRR002707, DRR002695, DRR002694, DRR002693	Illumina Genome Analyzer IIx	Single-end reads	Four biological replicates and three technical replicates	https://www.ncbi.nlm.nih.gov/bioproject/PRJDB2658
Shoot at four-leaf stage	Shoot5	SRR034588, SRR034581	Illumina Genome Analyzer	Single-end reads	Two technical replicates	https://www.ncbi.nlm.nih.gov/bioproject/PRJNA122435
Shoot apical meristem	SAM	SRR1999346, SRR1999345	Illumina HiSeq 2000	Paired-	Two technical replicates	https://www.ncbi.nlm.nih.gov/bioproject/PRJNA282397

at tillering stage				end reads		
Inflorescence meristem at booting stage	IM	SRR1999347,SRR1999348	Illumina HiSeq 2000	Paired-end reads	Two technical replicates	https://www.ncbi.nlm.nih.gov/bioproject/PRJNA282397
Stem at the 3-leaf stage	Stem	SRR1182462, SRR1182461, SRR1182460, SRR1182459, SRR1182458, SRR1182457	Illumina HiSeq 2000	Paired-end reads	Two biological replicates and three technical replicates	https://www.ncbi.nlm.nih.gov/bioproject/PRJNA239935
Internode at dough stage	Internode	SRR1174190	Illumina HiSeq 2000	Paired-end reads	No	https://www.ncbi.nlm.nih.gov/bioproject/PRJNA238962
Leaf at 7 day	Leaf1	SRR576932, SRR576931	Illumina Genome Analyzer II	Single-end reads	Two technical replicates	https://www.ncbi.nlm.nih.gov/bioproject/PRJNA176306
Leaf at 14 day	Leaf2	SRR358794, SRR358793, SRR358792, SRR358791	Illumina Genome Analyzer II	Single-end reads	Two biological replicates and Two	https://www.ncbi.nlm.nih.gov/bioproject/PRJNA149055

					technical replicates	
Leaf at tillering stage	Leaf3	SRR1298433, SRR1298432, SRR1298431, SRR1298430, SRR1298429, SRR1298428	Illumina HiSeq 2000	Single-end reads	Three treatments (well-watered, 1d drought stressed, and 3d drought stressed) and two replications	https://www.ncbi.nlm.nih.gov/bioproject/PRJNA248474
Leaf at heading stage	Leaf4	DRR001045, DRR001041, DRR001034, DRR001027	Illumina Genome Analyzer IIx	Single-end reads	One mix and three biological replicates	https://www.ncbi.nlm.nih.gov/bioproject/PRJDA67119
Lamina joints at heading stage	Joint	SRR976168	Illumina Genome Analyzer	Single-end reads	No	https://www.ncbi.nlm.nih.gov/bioproject/PRJNA218874
Leaf sheath at tilling stage	Sheath	SRR1562076, SRR1562075, SRR1562074	Illumina HiSeq 2000	paired-	Three biological replicates	https://www.ncbi.nlm.nih.gov/bioproject/PRJNA259819

				end reads		
Panicle at flowering stage	Panicle1	DRR001049, DRR001042, DRR001035, DRR001028, SRR037743	Illumina Genome Analyzer IIx	Singl e-end reads	Three biological replicates	https://www.ncbi.nlm.nih.gov/bioproject/PRJDA67119
Panicle at heading stage	Panicle2	DRR001048, DRR001041, DRR001034, DRR001027	Illumina Genome Analyzer IIx	Singl e-end reads	Three biological replicates	https://www.ncbi.nlm.nih.gov/bioproject/PRJDA67119
Panicle at booting stage	Panicle3	SRR037725, SRR924689, SRR1824323	Illumina Genome Analyzer	Singl e-end reads	Three technical replicates	https://www.ncbi.nlm.nih.gov/bioproject/PRJNA117345
Panicle at filling stage	Panicle4	SRR037745	Illumina Genome Analyzer	Singl e-end reads	No	https://www.ncbi.nlm.nih.gov/bioproject/PRJNA117345
Spikelet at booting stage	Spikelet	SRR342355, SRR517607	Illumina HiSeq 2000	Singl e-end reads	Two technical replicates	https://www.ncbi.nlm.nih.gov/bioproject/PRJNA147541

Grain at milk stage	Grain1	SRR1618548	Illumina HiSeq 2000	Paired-end reads	No	https://www.ncbi.nlm.nih.gov/bioproject/PRJNA262032
Grain at mature stage	Grain2	DRR001051, DRR001044, DRR001037, DRR001030	Illumina Genome Analyzer IIx	Single-end reads	Two technical replicates and three biological replicates	https://www.ncbi.nlm.nih.gov/bioproject/PRJDA67119
Embryo at milk stage	Grain3	SRR771504, SRR771501	Illumina Genome Analyzer IIx	Single-end reads	Two technical replicates	https://www.ncbi.nlm.nih.gov/bioproject/PRJNA192671
Aleurone at mature stage	Grain4	SRR946865, SRR946864, SRR946863	Illumina HiSeq 2000	Single-end reads	Three technical replicates	https://www.ncbi.nlm.nih.gov/bioproject/PRJNA213797
Grain endosperm at milk stage	Grain5	SRR771507, SRR771500	Illumina HiSeq 2000	Single-end reads	Two biological replicates	https://www.ncbi.nlm.nih.gov/bioproject/PRJNA192671
Grain endosperm	Grain6	SRR2338869, SRR2338868, SRR2338867, SRR2338866	Illumina HiSeq 2000	Paired-	Two biological replicates	https://www.ncbi.nlm.nih.gov/bioproject/PRJNA295115

at filling stage				end reads	and two biological replicates	
Pistil	Flower1	SRR1618547	Illumina HiSeq 2000	Paired-end reads	No	https://www.ncbi.nlm.nih.gov/bioproject/PRJNA262032
Anther	Flower2	SRR1618546	Illumina HiSeq 2000	Paired-end reads	No	https://www.ncbi.nlm.nih.gov/bioproject/PRJNA262032
Anther[sterile]	Flower3	SRR1982831, SRR1975011, SRR1974265, SRR1974208, SRR1974149, SRR1972541, SRR1972493, SRR1970544	Illumina HiSeq 2000	Paired-end reads	Five biological replicates and two technical replicates	https://www.ncbi.nlm.nih.gov/bioproject/PRJNA281699
Ovule of flower	Flower4	SRR976338, SRR976337, SRR976336	Illumina HiSeq 2000	Single-end reads	Three technical replicates	https://www.ncbi.nlm.nih.gov/bioproject/PRJNA218883
Pollens of uninucleate microspores	Pollen1	ERR035782	Illumina Genome Analyzer	Single-end reads	No	

Bicellular pollen	Pollen2	ERR035777	Illumina Genome Analyzer	Single-end reads	No	https://www.ncbi.nlm.nih.gov/bioproject/PRJEB2564
Tricellular pollen	Pollen3	ERR035781	Illumina Genome Analyzer	Single-end reads	No	https://www.ncbi.nlm.nih.gov/bioproject/PRJEB2564
Pollen at vegetative cell	Pollen4	SRR976343, SRR976342, SRR97635	Illumina HiSeq 2000	Single-end reads	Three technical replicates	https://www.ncbi.nlm.nih.gov/bioproject/PRJNA218883
Sperm	Pollen5	SRR976341, SRR976340, SRR976339	Illumina HiSeq 2000	Single-end reads	Three technical replicates	https://www.ncbi.nlm.nih.gov/bioproject/PRJNA218883
Coleoptiles, Radicals, Shoot, and Tillering leaf	Mix1	SRR527810	454 GS FLX Titanium	Single-end reads	No	https://www.ncbi.nlm.nih.gov/bioproject/PRJNA171494
Anther,Pistil and Grouting Embryo	Mix2	SRR527811	454 GS FLX Titanium	Single-end reads	No	https://www.ncbi.nlm.nih.gov/bioproject/PRJNA171494
