# Supplementary material for: Genome-Wide Identification and Expansion Patterns of SULTR Gene Family in Gramineae Crops and Their Expression Profiles under Abiotic Stress in Oryza sativa
Source: Genes (Basel). 2021 Apr 23;12(5):634. doi: 10.3390/genes12050634 (PMC8146379; doi:10.3390/genes12050634)
Supplement: Supplementary file 1 [file genes-12-00634-s001.zip › Supplementary table 2.pdf]

Supplementary Table 2: List of oligonucleotide primers used for qRT-PCR analysis

S.No.	Sulphate transporter	Forward	Reverse
1.	OsiSULTR1	5'-CTTCACCACCAAGACCGACA-3'	5'-AAGACGCATCCTACGACGAC-3'
2.	OsiSULTR2	5'-AGGGCTAATAGTGGATTTCCTCTC-3'	5'-GCAGTTTCTTTCTCAGGTGCTTAC-3'
3.	OsiSULTR3	5'-GGGAACATCCCCAACACGAT-3'	5'-GATCCTCTCACGCAGGTAGC-3'
4.	OsiSULTR4	5'-GCATCCGGAGAACGTCTTCA-3'	5'-TCCGCTCTTGTCGCATAGAC-3'
5.	OsiSULTR5	5'-CAAGACCGCCATCATCTGC-3'	5'-TCCTTGTTCCCGTCGAGTTT-3'
6.	OsiSULTR6	5'-AGGACCAGCCGATGTCAAAG-3'	5'-CCAGCAAGCTTCGCATAACC-3'
7.	OsiSULTR7	5'-GATAAGCCTTACGGAAGCG-3'	5'-TCATTGAACCGACCACATT-3'
8.	OsiSULTR8	5'-GAACCAAACAAAGTGAACTCCATT-3'	5'-AGCATCATTCGCTCGTTGTATCTT-3'
9.	OsiSULTR9	5'-CAGGTGATGCACTCCGTCTT-3'	5'-AGCTTTGCTGACGAACGAGA-3'
10.	OsiSULTR10	5'-TCCCAATCTTTGAGTGGGGC-3'	5'-CCCGATATCCTGGGGAATGC-3'
11.	OsiSULTR11	5'-GTTATACACAGGCTTTGTCCCGCT-3'	5'-TCAAGCATTCTAATACACCAACCA-3'
12.	OsiSULTR12	5'-CCAATTCAGGCTACCTGCGA-3'	5'-CTTCCAACATCTTCGTGCCG-3'
13.	Actin	5'-CTCGTCTGCGATAATGGAACTGGT-3'	5'-TGGGGTACTTGAGGGTCAAGATAC-3'
