# Supplementary material for: Genome-Wide Identification and Expansion Patterns of SULTR Gene Family in Gramineae Crops and Their Expression Profiles under Abiotic Stress in Oryza sativa
Source: Genes (Basel). 2021 Apr 23;12(5):634. doi: 10.3390/genes12050634 (PMC8146379; doi:10.3390/genes12050634)
Supplement: Supplementary file 1 [file genes-12-00634-s001.zip › Supplementary table 4.pdf]

Supplementary Table 4. Gramineae SULTR gene numbers in different clades

| Clade | Bd | Hv | Ob | Og | Osj | Osi | Or | Sb | Si | Zm | Total |
|---|---|---|---|---|---|---|---|---|---|---|---|
| I | 1 | 1 | 2 | 2 | 3 | 2 | 2 | 1 | 1 | 1 | 16 |
| II | 2 | 2 | 2 | 1 | 2 | 2 | 2 | 1 | 2 | 1 | 17 |
| III | 2 | 3 | 2 | 2 | 2 | 2 | 2 | 2 | 2 | 2 | 21 |
| IV | 2 | 2 | 2 | 3 | 3 | 3 | 2 | 3 | 4 | 2 | 26 |
| V | 3 | 3 | 3 | 3 | 3 | 3 | 2 | 3 | 3 | 4 | 31 |
