# Supplementary figures and images for: Genome-Wide Identification and Expansion Patterns of SULTR Gene Family in Gramineae Crops and Their Expression Profiles under Abiotic Stress in Oryza sativa
Source: Genes (Basel). 2021 Apr 23;12(5):634. doi: 10.3390/genes12050634 (PMC8146379; doi:10.3390/genes12050634)

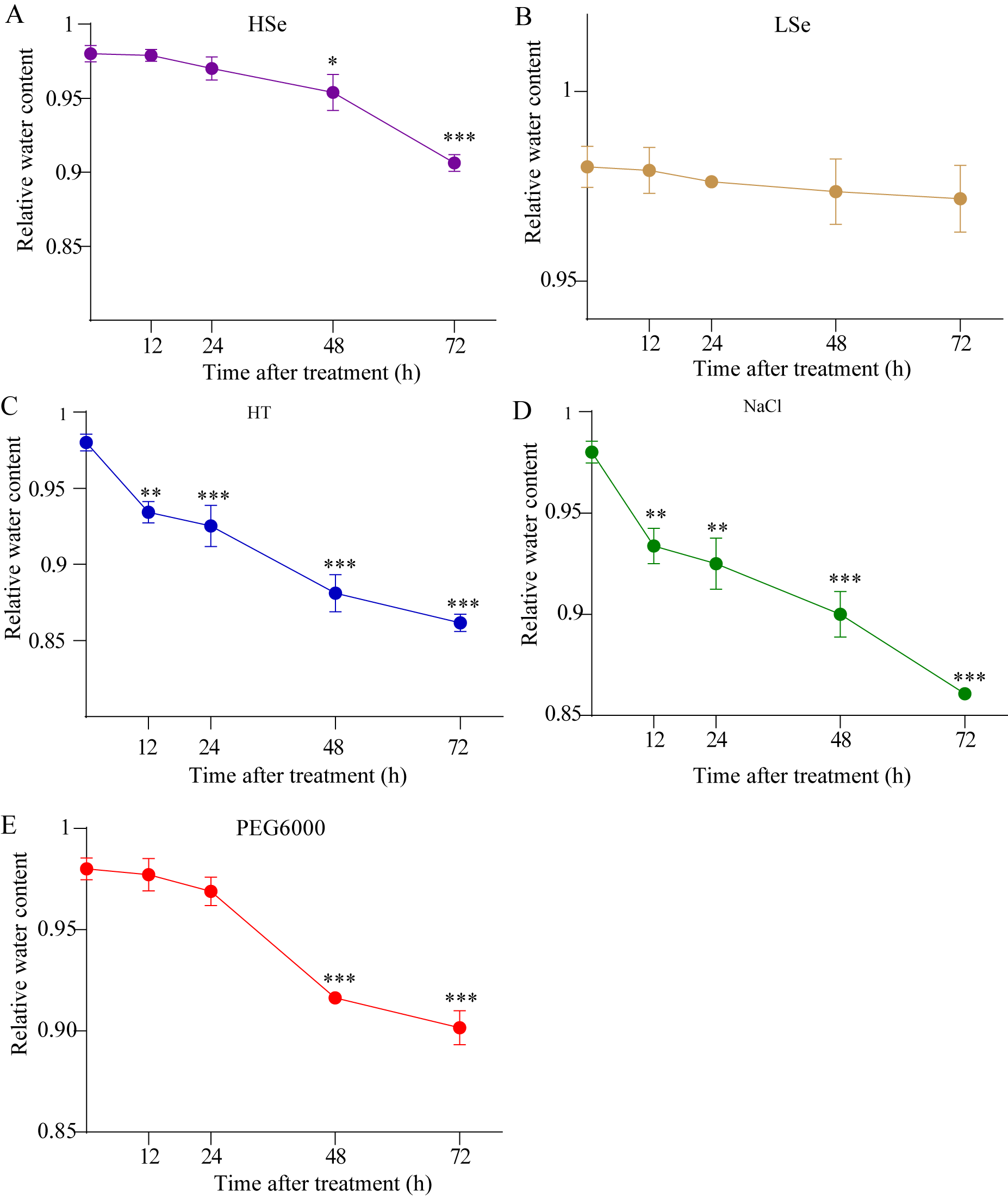

Supplement: Supplementary file 1 [file genes-12-00634-s001.zip › Supplementary Figure 1.tif]
